# Perceptions of telecare training needs in home healthcare services: a focus group study

**DOI:** 10.1186/s12913-017-2098-2

**Published:** 2017-02-23

**Authors:** Veslemøy Guise, Siri Wiig

**Affiliations:** Department of Health Studies, University of Stavanger, Kjell Arholms gate, 4036 Stavanger, Norway

**Keywords:** Telecare, Vocational training, Training needs, Healthcare professionals, Home healthcare, Focus groups

## Abstract

**Background:**

The implementation and use of telecare requires significant changes to healthcare service organisation and delivery, including new ways of working for staff. Competency development and training for healthcare professionals is therefore required to enable necessary adaptation of clinical practice and ensure competent provision of telecare services. It is however unclear what skills healthcare staff need when providing care at a distance and there is little empirical evidence on effective training strategies for telecare practice. Training should however emphasise the experiences and preferences of prospective trainees to ensure its relevance to their educational needs. The aim of this study was to explore healthcare professionals’ perceptions of training related to the general use of telecare, and to identify specific training needs associated with the use of virtual visits in the home healthcare services.

**Methods:**

Six focus group interviews were held with a total of 26 participants working in the home healthcare services in Norway, including registered nurses, enrolled nurses, physiotherapists, occupational therapists, social workers, health workers, and healthcare assistants. The data material was analysed by way of systematic text condensation.

**Results:**

The analysis resulted in five categories relevant to telecare training for healthcare professionals: Purposeful training creates confidence and changes attitudes; Training needs depend on ability to cope with telecare; The timing of training; Training must facilitate practical insight into the patients’ perspective; and Training content must focus on the telecare process. Findings are discussed in light of implications for the form and content of a training program for healthcare professionals on how to undertake virtual home healthcare visits.

**Conclusion:**

Appropriate preparation and training for telecare use is important for healthcare professionals and must be taken seriously by healthcare organisations. To facilitate the knowledge, skills and attitudes required for new ways of working and enable quality and safety in telecare practice, staff should be provided with training as part of telecare implementation processes. Telecare training should be hands-on and encourage an overall patient-centred approach to care to ensure good patient-professional relationships at a distance.

## Background

Telecare is the use of information and communication technologies (ICTs) that enable healthcare professionals to remotely care for and support individuals living in their own home. The implementation of telecare in healthcare services requires changes to service organization and delivery, including new ways of working for healthcare professionals [1–3]. Changes in the organization, modes and places of delivering care are not always compatible with conventional clinical practices and can be seen as a threat to traditional healthcare work and professional roles and responsibilities [4–7]. The use of telecare technologies in home healthcare services may well necessitate adjustments to practice and a reconsideration of the accepted understandings, knowledge and skills needed to know and care for patients [5], including changes to staff attitudes and the culture of home healthcare services [3, 8–10]. In this regard, staff training is widely viewed as fundamental to successful telecare implementation, to result in safe and effective telecare practice [1, 11–19]. Education and training for telecare practice is however an emergent research field with few empirical examples reported in the literature [20]. Knowledge on healthcare professionals’ telecare training needs and experiences is therefore scarce.

### The importance of training for telecare practice

It is increasingly recognised that specialised competencies are needed among healthcare professionals for sound telecare practice [11, 19, 21–23]. Conversely, an absence of the knowledge and skills needed to use and promote telecare is regarded as detrimental to the patient safety of telecare [24] and its sustained adoption in healthcare services [1, 13]. Targeted staff training is therefore important to ensure the competency of telecare practitioners and the safety of services, for example by facilitating standardized working practices in the delivery of telecare [13, 25]. Training has furthermore been found to have positive effects on user confidence and attitudes to ICTs in healthcare [26–28]. One of the biggest barriers to the sustained implementation of telecare are the negative attitudes of healthcare professionals [17, 19, 29, 30], including a lacking readiness to adapt to change and adopt innovation [9]. Raising awareness and building confidence through training can potentially change attitudes and help motivate healthcare professionals to adopt telecare competencies as part of their clinical practice skills [12, 14, 23, 31].

### Telecare training initiatives for healthcare professionals

Despite the evidence of the importance of training for telecare practice and repeated calls for a minimum standard of required competencies and training for telecare practitioners [21, 32], telecare training is often found to be inadequate or altogether lacking [3, 7, 10, 19, 24, 33, 34]. Rather than formal training, therefore, many healthcare professionals are instead learning how to undertake telecare practice on-the-job [35]. In line with these findings, there is a dearth of published research on formal telecare training interventions for healthcare professionals. A review by Basu and colleagues [20] on pedagogical and professional development models related to the use of telecare applications identified only ten training courses, of which only two were for healthcare professionals working in home healthcare services. While training initiatives driven by the practice field may certainly exist, the lack of published telecare training research means there is little empirical evidence available on the characteristics of effective training practices and how best to adapt practice to engage with new technologies [5, 19, 20]. There is however consensus on important considerations in the design, development and delivery of training programs, including training for telecare, to best achieve successful implementation and transfer of learning in practice [20, 36].

### Analysis of training needs to guide the design and delivery telecare training

Conducting a thorough training needs analysis is a fundamental first step in the systematic design, development, and delivery of training programs [37]. The aim of such an analysis is to ascertain factors that can significantly impact the delivery and outcomes of training [36]. This includes examination of the organizational context; pinpointing the characteristics and requirements of the job-tasks concerned; and identifying who the trainees are and what their characteristics, competencies, and training needs are. The outcomes of the analysis can be used to establish training objectives and intended learning outcomes, and to set standards of performance during training [37]. The outcomes can also inform the course content, educational methods and how the training is delivered, as well as the development of a plan for implementation and evaluation of the training initiative [20, 36, 37].

Analysis of the organisational context relates to such aspects as the strategic prioritization of training and the availability of resources and support [37], in addition to consideration of various organisational constraints on telecare initiatives, including the work environment, communication infrastructure and connectivity issues [20, 26]. Analysis of job-tasks includes a description of essential work characteristics and content, and an overview of the resources and conditions necessary for high quality job performance [36]. Importantly, this entails identification of the various specialised competencies telecare practitioners need to undertake their work [20, 38]. Finally, training cannot be effective unless it meets the needs of trainees [36, 39] and their readiness for learning [23]. An investigation of trainee characteristics, competencies, preparedness and educational requirements is therefore at the crux of the training needs analysis, to ascertain if they have the requisite attitudes and capabilities to undertake intended work tasks, and assess their motivation to learn and participate in training [36]. These are factors likely to impact not only internal learning processes, but also the practical application of new knowledge and skills [20, 26, 37].

Exploring the needs, preferences and experiences of prospective trainees has been advocated as a means of informing the design and delivery of telecare training initiatives [13, 26, 38]. To date, there has been little research on healthcare professionals’ telecare training needs and more studies are therefore needed to ensure appropriate preparation for new ways of working and foster sound telecare practice [23, 40].

## Aims

To address the lack of research on telecare training, the aims of this study were to

- explore perceptions of training related to the general use of telecare among healthcare professionals working in the home healthcare services, and
- identify healthcare professionals’ specific training needs associated with the use of virtual home healthcare visits.

The following research questions will be addressed:

1. *How is telecare training and the need for telecare training perceived by healthcare professionals in the home healthcare services?*
2. *What is the desired form and content of training for healthcare professionals intending to participate in virtual visits in the home healthcare services?*

## Methods

### Research design and context

The study described here was undertaken as part a five-phase action research project [41] called *Safer@Home - Simulation and training* [42]. The overall objective of the *Safer@Home* project was to develop, test, and evaluate a simulation-based vocational training program to prepare healthcare professionals from two municipal home healthcare services in Norway to conduct safe, high quality virtual visits. Virtual visits involve real-time audio-visual communication between healthcare professionals and patients through a secure video communication system and clinical uses include assessment of health status, monitoring of medication routines, and demonstration or supervision of procedures [43]. At the time of this study, the particular technological solution to be used had not been decided upon. The study was part of a training needs analysis [37] undertaken in the two organizations involved to guide the design and delivery of the simulation-based training program and ensure that the training objectives and associated training content were relevant to trainees’ needs.

Focus group interviews were used to explore healthcare professionals’ perceptions of telecare training and identify training needs associated with the use of virtual home healthcare visits. The focus group method is a useful data collection technique when the aim of the research is to explore attitudes, experiences, beliefs and concerns, as this approach taps into wide frameworks of understanding [44, 45] by emphasising group interaction and discussion [46]. Focus groups are also recommended when examining staff responses to organisational changes [45], such as the implementation of virtual visits in home healthcare services. In addition to the findings from the focus group study, training objectives were informed by various recommendations from the literature on the use of telecare in home healthcare services [11, 19, 20, 22–24, 43], as well as by a study on older patients’ experiences with virtual visits [47]. See Wiig et al. [42] for the for the full *Safer@Home* study protocol, and Guise & Wiig [48] for further detail on how the simulation-based telecare training program for home healthcare professionals was developed.

### Study sample

A total of six focus group interviews took place with altogether 26 participants, 23 women and three men, working in four different home healthcare or sheltered housing services in the two municipalities intending to pilot the use of virtual home healthcare visits. 18 participants were from Municipality A and eight were from Municipality B. A purposive sampling strategy was used to enable inclusion of a cross-section of the health and social care professionals working in the home context. There were seven registered nurses, four enrolled nurses, three physiotherapists, five occupational therapists, three social workers, one care worker, one social educator, one health worker, and one care assistant. Participants’ average age was 39 years (a range of 24–59 years), while the average total work experience was 13.75 years (a range of 1–37 years). Only a small minority of two participants had prior experience using video communication technology (Skype) for work.

### Data collection

The data collection was conducted according to an agreed study protocol [42] over a five-month period during 2013 and 2014, and was undertaken by a multi-disciplinary research team made up of health services researchers, healthcare educators, and one representative from municipal healthcare services. In order to take advantage of homogeneity, shared experiences, and existing group dynamics [49, 50], each focus group consisted of the professional groupings who usually work together in the included services: three groups with different constellations of registered nurses, enrolled nurses, healthcare workers and care assistants from home healthcare services, one group of physiotherapists from physiotherapy services, one group of occupational therapists from occupational therapy services, and one group of social care workers and social educators from sheltered housing services.

Due to the practicalities of taking clinical staff out of practice to participate in research, each of the six groups met only once, with each interview lasting between 90 and 120 min. Author VG moderated all six focus groups. One or two other members of the research team acted as observers in different focus groups, taking notes on group dynamics, atmosphere, and participant relations [50]. The degree of participation from the moderator and observers varied across the groups, though in the main, VG facilitated the group discussion while the observers occasionally followed up with additional questions and clarifications. The same predetermined set of topics was covered in all focus groups and included questions on telecare and technology experience; thoughts on implementation of virtual visits in home healthcare services; implications of using virtual visits; and training needs. Participants did not see the list of topics prior to the interviews, to reduce the risk of any predetermined responses and increase the chance of open focus group discussions.

### Data analysis

All focus group interviews were tape-recorded and transcribed verbatim, and the data were analysed by way of systematic text condensation [51]. This approach involves the following steps: (1) establishing an overall impression of the data material and identifying preliminary themes; (2) identifying and sorting units of meaning into code groups; (3) condensing the contents of each of the coded groups into subgroups; and (4) summarizing the contents of each code group to generalize descriptions and concepts, in this case related to perceptions of training and training needs. Both authors contributed to the data analysis, in part together with the larger research team who all read focus group transcripts to get an overall impression of the full data material, as per step (1) above. This was followed by a one-day data analysis workshop attended by team members, during which the following five preliminary themes were identified: Technology experience; Attitudes to telecare; Prerequisites for undertaking virtual visits; Implications of using virtual visits; and Training. This article reports on findings related to the theme ‘Training’. (An analysis of perceptions of quality and safety implications of using virtual visits has been published elsewhere [52].) All subsequent data analysis (steps 2–4 of the systematic text condensation process) pertaining to the ‘Training’ theme were undertaken by author VG, with input from author SW. The analytical process is shown in Table 1.

**Table 1:** Analytical process and categories

Meaning units (selected)	Subgroups	Categories
If you are going to have to use something new, it’s good to get the training you need to do so.	Importance of proper staff training	Purposeful training creates confidence and changes attitudes
‘It [training] is supposed to be appropriate – what are we going to find out about, what are we going to use it [telecare] for?	Training to fit purpose of tasks and technology	
That you are confident in what you are going to do after you have received that training. That you know you will master it [the new task].	Training creates confidence	
If everyone has the feeling of receiving good training, it’ll be a lot more effective, then you’ll change attitudes.	Training changes attitudes	
We have a big staff group, 50 to 60 people, and within it there are a lot of different attitudes and experiences.	Different staff have differing abilities	Training needs depend on ability to cope with telecare
Personally I feel that I would not need that much [training]. I think it would probably be enough with a few hours.	Confidence in own ability to cope	
We have mostly elderly users over 70 years old, right. I do not think they can manage to use it [technology]. It will be difficult for them.	Difficult for elderly to cope with technology	
They [the patients] are so astute many of them, they manage with all kinds of stuff [technologies] like that.	Some elderly can cope with technology	
I think that the elderly now gradually appearing, they are more accustomed to technology and that makes it easier to introduce. Those who are old now, they have never had it in their hands. So it [implementation] will probably become easier.	The elderly of the future will cope better	
The gap is big in the need for training. Some take things on very quickly. They are so accustomed to technology that they think “really, we need to sit here for 3 h and listen to this?” Some need a lot of training, while for others a little user manual will be enough.	Individual users have individual training needs	
One must be very adept at assessing, when one is out there, will the person be able to use this, with training?	Assessment of patient ability to cope	
We are given training, but then the new system is not up and running until 3–4 months later, and by then we’ve forgotten it all. [Implementation] has to be straight after we’ve had the training, or else it needs to wait.	Training must not be given too far in advance of active use	The timing of training
And that there is time for it [training]! Not least. That was an error that was made here, that there was not enough time set aside.	There must be enough time for training	
[Training] may well require some time away from other things, from everyday services. And I think we already have too much of all types of different things that we do.	Training should not take up too much time	
The best way to learn it is to use it. We need training where we get to try the equipment ourselves, to know how it works, [to see] what gives the most beneficial effect.	Learning through practical use	Training must facilitate practical insight into the patients’ perspective
It is important that we get to try it [virtual visits] as service users. So that we know what they have to deal with.	Understanding the user perspective	
[Simulation] would be very educational and informative for everyone.	Positive to the use of simulation	
The practical things, both for us and for the one at the other end. How to turn it on and off and how to make a call. The basics.	Technical skills	Training content must focus on the telecare process
How do you communicate? What is smart to say and what is not smart to say? What questions should you ask? This will depend somewhat on who you are talking to.	Communication techniques	
I think also creating a little awareness about the ethical aspects, in relation to it being through a screen and, well, how there may be others [present with the patient] who can hear.	Ethical aspects	
I think it is important for the user that it is someone who they trust and can have a chat with. That they can make mistakes but feel safe.	How to train and support patients	

## Results

The analysis resulted in five categories, two of which correspond with research question 1 on general perceptions of telecare training and training needs. Participants talked about the perceived purpose of training for telecare, as well as their views on different end-users’ abilities to cope with telecare and the implications of this for training. Their own perceived training needs were closely linked to patients’ needs and abilities. Three categories correspond with research question 2 on perceptions of the specific type of training needed to conduct virtual home healthcare visits. Participant preferences were expressed in terms of views on the optimal timing of training, as well as reflections on the desired form and content of training. The findings are described in more detail in the following. See Table 1 for illustrative quotes from each analytical category.

### Purposeful training creates confidence and changes attitudes

Receiving proper training was important for healthcare professionals faced with new ways of working. Participants’ views on the importance of suitable training were often based on previous poor training experiences. They told several anecdotes to illustrate the futility of insufficient or lacking training resulting in new technologies in the workplace not being used as planned or not being used at all. Participants therefore wanted ‘good’ training that is purposeful and appropriate to the specific needs triggered by the tasks and technological tools in question. Participants felt the main purpose of training was to create confidence among staff about working in a way that is new and unfamiliar. Furthermore, good training should be able to change staff attitudes to telecare, where needed. Having access to proper, purposeful training that could foster attitude changes and build confidence was seen to have positive implications beyond the individual staff members receiving the training.

### Training needs depend on ability to cope with telecare

The issue of healthcare professionals’ and patients’ abilities to cope with telecare was a major topic of discussion in all the focus groups. Participants were very aware that different healthcare staff will have different interests and capabilities in regards to working in new ways with unfamiliar technical tools. They realized that due to this variety in abilities, different colleagues will have differing training needs. In particular, it was felt that older staff members may have more pronounced training needs than younger colleagues due to a lack of prior experience with technology, as well as an aversion to change. In contrast, participants had confidence in their own ability to master new ways of working. They felt that with the right training and support, things would fall into place little by little, ‘as usual’.

In addition to the staffs’ coping abilities, participants talked at length about service users’ coping abilities related to the use of telecare. They recognised that the use of virtual home healthcare visits would be very much contingent on their patients’ ability to use the video communication technology. Participants’ views on the abilities of older service users to cope with new technologies and hence their training needs, were very divergent, making this the most contentious topic within several of the groups. Again, concerns here were related to the different levels of interest and prior skills among intended users of telecare, and how to best accommodate these diverse needs during training. While it was acknowledged that service users cope differently with technology, many argued strongly that most elderly users have big difficulties coping with technology and that it is hard for them to learn new things. Many participants felt that the only way older users will be able to cope, is if the technology is very simple and familiar, though others argued it would not be possible even then. Many did not see training as being of use to these patients either.

In contrast to these negative views, some participants argued that many elderly are both interested in and able to learn new skills and how to cope with new technologies. In general, however, participants felt more positive about the prospect of coming generations of patients being better able to manage new technologies than today’s older people. They therefore reasoned that the implementation of telecare in home healthcare services would become a lot easier in the future.

In acknowledging these differing interests and prior skills among their colleagues and patients, participants felt it was important that training for both healthcare professionals and service users should be tailored to accommodate a big variety of resultant training needs among all telecare users. Due to the differing prerequisites for coping with telecare and the perceived variety in training needs, participants suggested arranging extra staff training sessions for those who need it, and noted the importance of creating a low threshold for being able to ask questions and express uncertainty. They furthermore suggested undertaking ongoing evaluations of patients’ abilities to cope and individual assessments of the amount of training and support each patient would need to participate in telecare activities.

### The timing of training

Participants were concerned about the right timing of training, which contributed to its perceived quality. Many participants shared examples from previous experiences where training had not been organized in an optimal way. Firstly, they expressed dissatisfaction with training that was given too far ahead of the active use of new technologies and systems. By the time they then needed to use new skills, they had forgotten how to do so. Receiving telecare training close in time to active use was thus of major importance for participants. Another crucial issue to do with the timing of training was that enough time was provided for training. Again, previous experiences had been poor, where too little time for training had resulted in suboptimal learning for staff. Some participants were however wary of having attendance at training add to already busy work schedules, taking time away from other, more urgent tasks.

### Training must facilitate practical insight into the patients’ perspective

Participants across all focus groups had very similar thoughts on the preferred form of training for virtual home healthcare visits. They were firm in that training should primarily be practical in nature, with emphasis on hands-on experience of what this new way of working entails. Among the reasons for wanting a practically-oriented form of training was being able to make mistakes before undertaking virtual visits with real patients. Another was to be able to get a good understanding of the service user perspective of participating in virtual visits. Simulation was seen as an apt educational approach for learning how to conduct virtual visits from a practical hands-on angle, while gaining the desired insight into the user perspective.

### Training content must focus on the telecare process

Participants voiced the need for broad training content focused on the entire process of conducting virtual visits, to maintain sound delivery of patient care according to individual healthcare needs. This included a desire not only for the relevant technical skills needed to work the equipment, but crucially also comprehensive insight into communication skills and techniques appropriate to delivering care at a distance. Several participants felt that the appropriate training content would also depend on the particular purpose and desired outcomes of using virtual visits with specific patients or patient groups. A focus on individual patients and their respective needs were anticipated to be especially important factors to consider in regards to how to apply appropriate communication techniques.

Some participants also expressed the need for training content related to the ethical aspects of undertaking virtual visits, such as how to uphold patient confidentiality, for example if the patient has a visitor present. Finally, participants were also interested in content on how to train and support patients in the use of virtual visits. This was because they regarded established relationships with service users as an important consideration in the delivery of patient training, mainly to ensure a sense of security regarding a new way of receiving care. Participants mainly envisioned this training taking place in the patient’s home.

## Discussion

As part of a wider training needs analysis, a focus group study was conducted to explore healthcare professionals’ experiences, attitudes and training needs associated with the intended implementation of virtual visits as part of municipal home healthcare services. In the following, the findings are discussed in light of implications for the form and content of a training program for healthcare professionals in how to conduct virtual home healthcare visits.

### Perceptions of telecare training and training needs

Receiving proper, purposeful and needs-oriented training is important to healthcare professionals expected to undertake the new ways of working implied by the implementation of telecare. This is in contrast to the informal learning that a majority of healthcare professionals report in regards to telecare practice [35]. The importance of training was largely expressed in terms of its role in creating the necessary confidence to master new skills and foster the attitudes necessary to provide care at a distance and successfully adopt telecare practice [3, 12, 17, 30, 31]. Carter, Horrigan and Hudyma [23] note similar findings, in that telecare training was regarded as important in fostering practitioner confidence and a positive attitude towards the use of telecare in the delivery of healthcare services. As such, staff training should emphasise a positive view of telecare as a way of enabling and supporting, as opposed to replacing, existing professional competencies and care processes [14, 19].

Participants argued that training needs, and the depth and elaborateness of training, would depend on individual ability to cope with the telecare service in question. Most participants felt that they would be able to cope well with the new work tasks associated with virtual visits and thus would not need much in-depth training. They were however much less confident in their older colleagues’ ability and willingness to engage with healthcare technologies. Similar findings have been noted elsewhere [53]. Due to the perceived differences in coping abilities, participants suggested that telecare training should be tailored to individual needs and offer a variety of forms of learning support. The importance of facilitating healthcare professionals’ different attitudes and prerequisites for adopting telecare has also been noted by Vuononvirta and colleagues [54]. In response to a potential lack of prior knowledge among some healthcare professionals, therefore, it could be advantageous for telecare training to include basic information on the relevance and potential of telecare, along with explanations and demonstrations of how relevant technologies and services work [3, 12, 14, 20].

Participants also had a strong service user focus in their reflections on coping abilities and training needs related to the implementation and use of virtual home healthcare visits. Though some expressed confidence in elderly patients’ interest in receiving telecare services and their ability to engage in training, some of our findings also mirrored previous research where healthcare professionals voice considerable scepticism towards the technical interests and abilities of elderly patients [14, 55]. Some participants indeed argued that older patients would not be able to learn the skills needed to operate new telecare devices regardless of training interventions. These attitudes reflect commonly held myths about older people and technology, namely that they have a general disinterest in technology, a lacking ability to understand and use technological devices, and are both unmotivated and cognitively unable to learn how to use new devices [56]. Research has however shown that older patients are both willing and able to participate in virtual home healthcare visits and that they report positive user experiences [47].

Wandke, Sengpiel and Sönksen [56] argue that negative attitudes among staff need to be actively counteracted as they may prevent older people from engaging with technological aids. Clark and McGee-Lennon [14] note that such opinions likely indicate a lack of practitioner knowledge of the potential of telecare services and how to appropriately match services to possible users. Horton [12] too reports similar findings regarding nurses’ lack of knowledge and a resultant reluctance to refer patients to telecare services. Telecare training for healthcare professionals should therefore include information on the potential pros and cons of telecare services, while encouraging a patient-centred [13] understanding of the advantages that telecare can offer differing user groups, such as increased opportunities for self-care activities and patient empowerment [6, 12, 14, 19, 52]. Furthermore, healthcare professionals need knowledge on how to match patients with appropriate telecare tools, and, as was suggested by our participants, awareness and understanding of how to continually assess the applicability, relevance and effectiveness of given telecare services to patients’ individual situations and needs [10, 14, 30, 57].

### Training preferences for conducting virtual visits

Participants offered several ideas regarding appropriate strategies and content for training and competence development in how to undertake virtual visits. The timing of training was regarded as an important consideration. Training must not be given too far ahead of implementation and active use of new devices or systems, and equally important, enough time must be set aside for training for it to have optimal outcomes for staff. The scheduling of training close in time to when trainees are meant to practically apply what they have learnt is indeed important to reduce skill decay [37]. As regards the desired form of training, there was broad agreement that it should be hands-on and that it must facilitate practical insight into the service user perspective of partaking in virtual visits. This is in accordance with earlier literature, which states that educational methods applied to telecare training for healthcare professionals should encourage preparation for practice through practical hands-on experience [3, 13, 14, 20, 26, 38, 58].

One inherently hands-on approach to the development of knowledge and skills for practical application is simulation [59]. Study participants were generally very positive towards the prospect of using simulation as a training method to create knowledge and awareness of what virtual visits will be like in practice. Simulation allows training content to be easily tailored to differing objectives and desired outcomes associated with undertaking virtual visits in diverse home healthcare services [43], a training concern that some of the participants reflected on. While there is growing indication that simulation may be a valuable means of teaching telecare practice to qualified healthcare professionals [60] as well as nursing students [40, 61–63], the potentials in using simulation in vocational telecare training for home healthcare staff remain largely unexplored.

Finally, regarding training content, participants expressed a need for a varied, process-focused content such as relevant technical and ethical aspects of conducting virtual visits. Of particular concern, however, was being able to maintain good communication with patients. Participants thus expressed a need for training focused on adaptation of relevant communication skills and techniques, to compensate for the changes to the ways they perceive and interact with patients that are implied by the use of telecare services [5]. Training for undertaking virtual visits should therefore focus on advanced communication, assessment and critical thinking skills, all of which are related to creating and maintaining effective, trusting and supportive patient-professional relationships in a virtual environment [6, 23, 64].

### Limitations

This study has some limitations. As the focus for this study was telecare in the home healthcare setting, insight from related fields of practice such as telerehabilitation and telepsychiatry has not been consulted. A broader perspective, including discussion of the telerehabilitation and telepsychiatry literature, could be relevant for other studies with a broader scope or a comparative design focusing on training in diverse fields of practice. Future research on the telecare training needs of healthcare professionals may also consider looking at participant perceptions according to age or length of work experience, as well as potential variety of opinion according to professional groupings, as none of these aspects were covered in this study. Furthermore, this study did not examine how individual staff members’ readiness for change associated with telecare use affects their views on the change readiness of patients. This is something that other studies may wish to consider.

## Conclusion

The findings reported here support the assertion that telecare training is not simply about learning how to master new technologies. It is primarily about learning how to adjust to novel work processes and how best to adapt traditional professional roles and work practices to new ways of providing healthcare [3, 6, 16, 19, 20]. Telecare training for healthcare professionals must therefore be taken seriously by healthcare organisations, technology developers, and the research field. Staff should be provided with structured, needs-based training as part of telecare implementation processes, to facilitate the knowledge, skills and attitudes required for new ways of working and to ensure the quality and safety of telecare services [65, 66]. The outcomes of this study indicate that intended telecare providers want hands-on training to facilitate practical insight into virtual visits, including from the patient’s experience. In addition, they want training to encourage an overall patient-centred approach to care, focused on the adaptation of communication techniques that will allow for sound clinical assessments and preservation of safe clinical relationships at a distance.
